# Diet-driven cardiometabolic dysfunction as a potential amplifier of antimicrobial resistance: mechanistic evidence, gut resistome ecology, and dual-action nutraceutical interventions

**DOI:** 10.3389/fpubh.2026.1977386

**Published:** 2026-10-12

**Authors:** Michael Tosin Bayode, Bashirat Akinbami, Christianah Titilayo Oladele, Ayomide Faith Okiti, Esther Oluwadarasimi Oyedeji

**Affiliations:** 1 Department of Microbiology, School of Life Sciences, Federal University of Technology Akure, Akure, Nigeria; 2 Department of Biochemistry, School of Life Sciences, Federal University of Technology Akure, Akure, Nigeria; 3 Teddy Bioscience Laboratory, Ilara-Mokin, Nigeria; 4 Department of Microbiology, Adekunle Ajasin University, Akungba-Akoko, Nigeria; 5 Food Science and Agricultural Chemistry, School of Environmental and Agricultural Sciences, McGill University, Montreal, QC, Canada; 6 Department of Food Science and Technology, School of Agriculture and Agricultural Technology, Federal University of Technology Akure, Akure, Nigeria

**Keywords:** antimicrobial resistance, berberine, bioactive compounds, cardiometabolic disease, gut resistome, nutraceuticals, One Health, public health

## Abstract

Over the last 30 years, two population-scale health crises have emerged as the epidemic of diet-related cardiometabolic disease (obesity, type 2 diabetes, hypertension, and cardiovascular disease) and the spread of antimicrobial resistance (AMR) as a major cause of death globally. Typically, they are investigated and controlled independently of each other. AMR may be amplified by diet-induced cardiometabolic dysfunction, which is not a parallel but an upstream, under-recognized burden. We describe four convergent pathways that might contribute to the increased risk of resistance. Impaired host immunity leading to recurrent, difficult-to-treat infection and repeated antibiotic exposure; obesity-associated pharmacokinetic changes leading to subtherapeutic antibiotic concentrations in the system; diet-induced gut dysbiosis that increases the reservoir of antibiotic-resistance genes (the gut resistome); and a bidirectional loop in which cumulative antibiotic exposure itself seems to increase cardiovascular risk. We then placed functional foods, nutraceuticals, and specific bioactive compounds as dual-action levers that act on the metabolic driver and, for a subset of molecules, directly on bacterial fitness, virulence, or the resistome. The clearest single-molecule example is developed in the form of berberine, which provides metabolic correction through the AMPK pathway, while simultaneously providing FtsZ-targeting and efflux-modulating antibacterial activity. We propose a testable framework, the metabolic–AMR augmentation loop, and highlight the gaps in evidence for longitudinal and interventional studies. If this framework proves to be robust, then diet-induced metabolic dysfunction correction could become a viable AMR-mitigation strategy on its own and one not currently employed in existing stewardship programs.

## Graphical abstract

**Figure:**
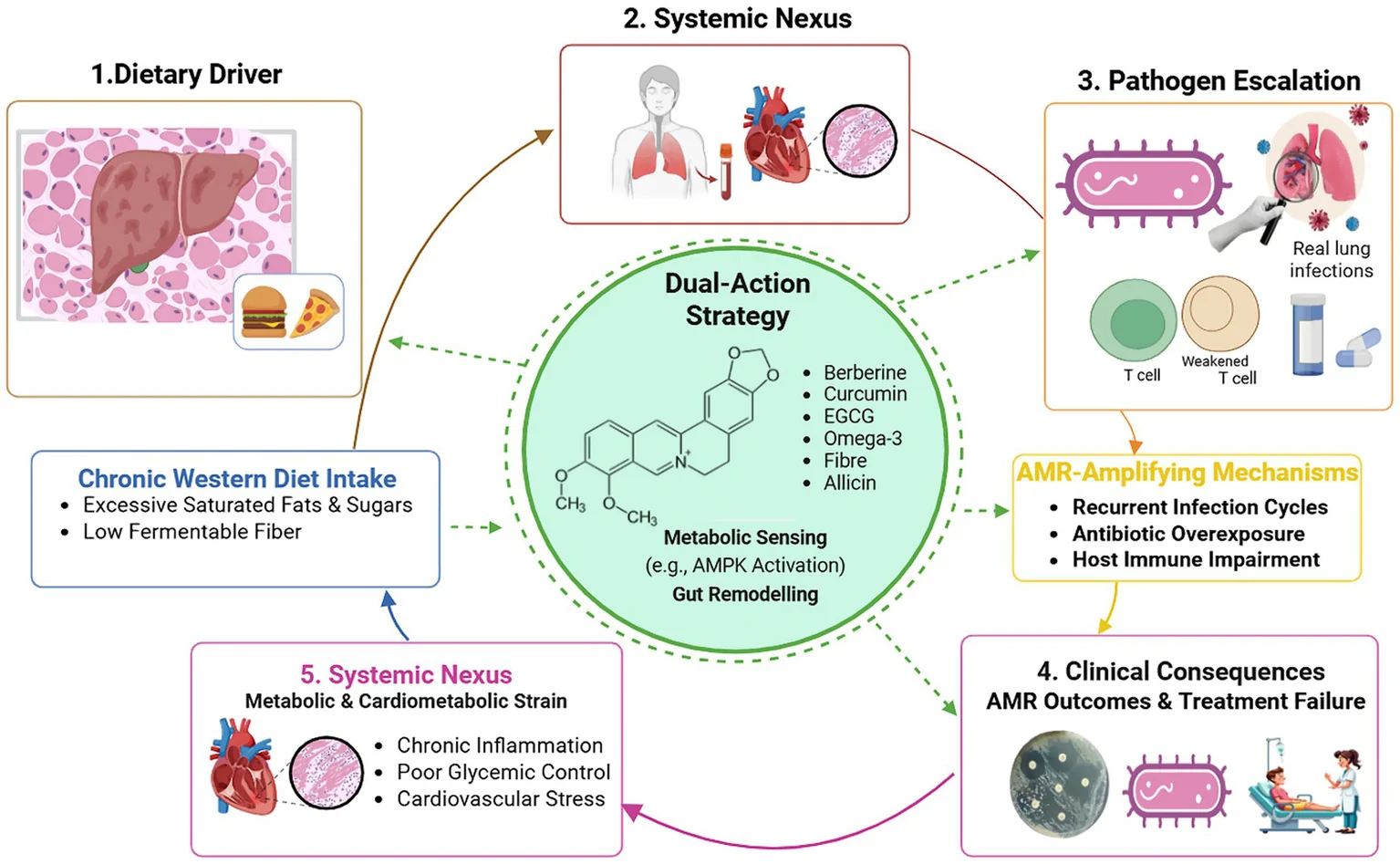

## 1 Introduction

The systematic reconstruction of the global burden of bacterial AMR in 2019 estimated 1.27 million deaths directly caused by bacterial AMR and was associated with an estimated 4.95 million deaths globally (1), making it one of the largest causes of death in the world. The force behind it is not waning: global use of antibiotics has continued to increase into the early 2020s and is expected to increase further if there is no strong stewardship (2). Proximal drivers of resistance include the volume and pattern of antimicrobial use, which is in turn driven by who is infected, the frequency of infection, and the effectiveness of treatment.

During the same period, the global diet has been changing to more energy-dense ultra-processed foods, high in saturated fat and refined sugar, and low in fermentable fiber, fruit, and vegetables (3–6). This dietary shift is the primary modifiable cause of obesity, type 2 diabetes (T2D), hypertension, and cardiovascular disease (CVD), which we refer to as cardiometabolic dysfunction. The nutritional epidemiology underpinning this has advanced, with large prospective cohorts consistently associating refined carbohydrate and processed meat consumption with metabolic and cardiovascular risks, and plant-rich patterns with protection (4, 5).

The analysis of these two epidemics is virtually always performed independently. AMR is seen as a microbiological and stewardship issue, and cardiometabolic disease is a chronic non-communicable disease (NCD) issue. There is not much systematic work on the intersection, that is, how a person’s metabolic state may affect their personal and community-level resistance. However, there are several biological contact points. All of these changes in metabolic function can impact immune competence, drug distribution, and the ecology of the gut microbial community, which in turn affects infection frequency, treatment success, and the size of the resident resistance-gene pool (7–9).

This review suggests a single hypothesis, diet-induced cardiometabolic dysfunction may be an upstream ecological and physiological amplifier of AMR via interwoven host–microbiome pathogen interactions, and that nutraceuticals that correct metabolic dysfunction could, as a downstream effect, act as “dual-action” interventions that ease resistance-selective pressure.

We aim to create a mechanistic link between metabolic diseases and AMR, list bioactive compounds that are likely to interact with both sides of this link, and outline a public health implementation and research strategy. We are very clear about our scope. This is a hypothesis-generating conceptual synthesis, not a pre-registered systematic review of the literature on diabetes–AMR and nutraceutical-AMR. We develop the argument from the best primary evidence we can find and call attention to where the evidence remains indirect, not simply pretending it does not exist.

### 1.1 The scope and literature base for the selective evidence

This is a conceptual synthesis, and not a systematic review, so we owe the reader an explanation of how the evidence for this synthesis was collected. We conducted a search of PubMed/MEDLINE, Scopus, Web of Science, and Google Scholar for English-language publications from 2000 to 2025, prioritizing the last decade because of the rapid pace of change in both the AMR and the cardiometabolic-disease literature. Search terms included the metabolic side (obesity, type 2 diabetes, hypertension, cardiovascular disease, metabolic syndrome, cardiometabolic dysfunction) and the resistance side (antimicrobial resistance, antibiotic resistance, gut resistome, antibiotic resistance genes), and each nutraceutical was discussed in section 6 separately. If a systematic review or meta-analysis was available for a particular claim, we used that over individual studies, and if there was only mechanistic, *in vitro*, or animal evidence, we have tried not to make too much of the citation. Where available, human and longitudinal studies were preferred, and preclinical and mechanistic studies were used primarily to provide biological plausibility, not as a replacement for clinical evidence. This process does not purport to be the reproducibility of a registered systematic review and does not claim to be one; it is our best attempt to be transparent about how an argument across this many fields was actually constructed.

## 2 Common dietetic upstream driver

### 2.1 The Western dietary transition

The modern Western dietary pattern is not characterized by any single nutrient but rather by a quality change in the food, characterized by an increase in refined starches, added sugars, and industrially processed products, replacing whole grains, legumes, fruits, and vegetables (4, 5). However, after decades of cohort and trial evidence, Willett et al. determined that the blanket recommendation to limit total fat has failed the test of time and that it is the quality of fat, carbohydrate, and the replacement of unsaturated plant fats with refined starch and sugar that is linked to poorer cardiometabolic outcomes (4, 5, 10). The relevance of this review is that the same dietary characteristics that most strongly promote metabolic disease low fiber, high simple sugar, high saturated fat are the ones that most strongly remodel the gut microbial community (3, 6, 7).

Mozaffarian (10) He conducted a thorough review of dietary and policy priorities and further clarified the situation by abandoning the previous single-nutrient accounting system. He contended that decades of focusing on specific targets, such as total fat and subsequently total carbohydrates, were misguided and led both science and policy to focus on the wrong targets, and that the evidence suggested that whole foods and total diet patterns were the targets to which to attend (10). If this is the case, minimally processed plant foods, whole grains, legumes, nuts, fish, and vegetable oils fall on the protective side. On the other hand, refined grains, added sugars, sugar-sweetened beverages, and processed meats are grouped together on the harmful side, with few exceptions in the macronutrient section (10). It is not any single nutrient, but rather the degree of industrial processing that becomes a major axis of food quality. As section 4 demonstrates, it is this axis that the gut resistome sorts along.

It is worth mentioning the food category that is on the bad side of the axis, which is closest to the AMR curve in the world. Monteiro and colleagues defined this category as ultra-processed food and drink products (Group 3), which are industrial formulations of cheap refined substances and additives, designed to be hyper-palatable, long-lasting and convenient for consumption, and are usually packaged as snacks (11). They compiled a list of ultra-processed products using Euromonitor sales data from high- and middle-income countries and found that these foods were already overrepresented in the diets of high-income countries and were rapidly gaining ground in middle-income countries, replacing traditional meals made from fresh and less-processed foods (11). They saw this change as more than a fad: the transnational food and snack producers they termed “Big Food” and “Big Snack” had developed their most profitable and heavily promoted product lines on these formulations. Thus, the market is now shifting toward ultra-processed foods (11). The relevance to our argument is straightforward. The dietary signature of Group 3, low fiber, high refined sugar, and high saturated and industrial fat, is the same signature that most effectively shifts the gut community to the low-diversity, facultative-anaerobe-rich state that carries and trades resistance genes (sections 2.3 and 4). Therefore, in addition to being an obesity narrative, ultra-processed dominance is, we argue, a reengineering of the ecological substrate on which the gut resistome develops on a population scale.

In Table 1, the NOVA groups are aligned with their cardiometabolic and gut-ecological signatures, with a clear explanation of why there is a step up in the processing gradient, which is a step toward a resistance-permissive gut.

**Table 1:** The NOVA food-processing classification and its cardiometabolic and gut-ecological correlates.

NOVA group	Representative foods	Processing hallmark	Cardiometabolic signal	Gut-ecological/Resistome relevance
Group 1—Unprocessed or minimally processed	Fresh and dried fruit and vegetables, legumes, whole grains, fresh meat and fish, eggs, plain milk and yogurt	None or minimal drying, chilling, fermentation, non-additive packaging; nothing added	Protective; the backbone of evidence-based patterns such as the Mediterranean and DASH diets (10)	Fermentable fiber feeds obligate anaerobes, raising short-chain fatty acids and lowering luminal pH conditions that suppress ARG-carrying Enterobacteriaceae (3, 26)
Group 2—Processed culinary ingredients	Vegetable oils, butter, sugar, salt, starches	Extracted or refined from Group 1 foods for use in cooking	Broadly neutral in normal culinary amounts; adverse in the excess that ultra-processing encourages (10)	Indirect; surplus refined sugar and starch lower the fermentable-fiber ratio that anaerobes depend on (3)
Group 3—Ultra-processed food and drink products	Soft drinks, packaged snacks and confectionery, reconstituted meat products, instant noodles and soups, sweetened cereals	Industrial formulation of refined substances plus additives; hyper-palatable, durable, ready-to-consume, often snack form (11)	Energy-dense and obesogenic; rising intake tracks obesity, type 2 diabetes, hypertension and cardiovascular disease (10, 11)	Low fiber with high fat and sugar drives loss of diversity and a bloom of facultative anaerobes, the community that most readily acquires, hosts and transfers resistance genes (3, 7, 26)

Group definitions and global dominance data follow Monteiro et al. (11); food quality and cardiometabolic grading follow Mozaffarian (10); the resistome column draws on the diet–resistome evidence synthesized in sections 2.3 and 4 (3, 7, 26).

### 2.2 Chronic inflammation and metabolic dysfunction

One of the hallmarks of diet-induced metabolic disease is chronic low-grade inflammation. Gut-derived lipopolysaccharide and other microbial products enter the circulation and lead to the chronic production of pro-inflammatory cytokines such as TNF-α, IL-1β, and IL-6 in excess adiposity and high-fat, high-sugar diets (3, 12). This “metaflammation” is not just a sign of metabolic disease, but it can actually impair the host defenses that decide whether the organism that colonized the host becomes an infection that can be treated. Chronic exposure to cytokines and oxidative stress damages innate immune effectors and disrupts epithelial barrier integrity, leading to the immune pathway described in section 3 (12).

Hotamisligil (12) He synthesized the immunometabolism field, providing this state with precise mechanistic grammar. He explained that immune and metabolic pathways share their core signaling machinery (the stress kinases JNK and IKKβ/NF-κB, sensing of saturated fatty acids by Toll-like receptor 4, and lipid and metabolite danger signals by the NLRP3 inflammasome), and that a chronic nutrient surplus maintains this shared machinery in a low-grade, maladaptive state (12). The picture that emerges is of an immune system permanently engaged in metabolic business—not in reserve for real pathogens—the same wiring that causes insulin resistance also compromises the fidelity of the acute antimicrobial response (12). Erosion is the mechanistic starting point for the immune pathway described in section 3, and Table 2 shows the key nodes of this metaflammatory pathway and their effects on host defense.

**Table 2:** The possibility of diet-induced inflammatory signaling pathways and their potential implications for antimicrobial host defense.

Signaling node/Mediator	Diet-derived trigger	Metabolic consequence	Plausible consequence for AMR-relevant host defense
JNK and IKKβ/NF-κB stress kinases	Saturated fatty acids, nutrient surplus, endoplasmic-reticulum and organelle stress	Serine phosphorylation of insulin-receptor substrates and sustained pro-inflammatory transcription, driving insulin resistance (12)	A constant low-grade cytokine tone reallocates and desensitizes immune signaling, blunting the sharp, timely response a new infection demands
Toll-like receptor 4 (TLR4)	Saturated fatty acids and gut-derived lipopolysaccharide (“metabolic endotoxemia”)	Chronic innate activation with elevated TNF-α and IL-6 output (12)	Persistent innate engagement alongside a leakier epithelial barrier favors recurrent colonization and harder-to-clear infection
NLRP3 inflammasome	Ceramides, palmitate, cholesterol crystals, uric acid	Caspase-1-driven maturation of IL-1β with inflammation of adipose tissue and pancreatic islets (12)	An IL-1β-skewed milieu distorts the coordinated antimicrobial program, favoring inflammation over efficient pathogen killing
Adipose-tissue macrophages/TNF-α	Excess adiposity and high-fat feeding	M1 macrophage polarization; TNF-α couples obesity to systemic insulin resistance (12)	Macrophage effort redirected to metabolic surveillance leaves less capacity for pathogen clearance at mucosal and wound surfaces

Node definitions, diet-derived triggers and metabolic consequences follow Hotamisligil’s immunometabolism synthesis (12); the final column is this review’s interpretive bridge to the immune pathway of section 3 and is provided as a possible mechanism.

### 2.3 Diet-induced gut dysbiosis

Diet is one of the most influential factors affecting the composition of the gut microbiome (3, 6). In a controlled gnotobiotic model, a switch to a Western high-fat, high-sugar diet led to a rapid, reproducible restructuring of the distal gut community: a bloom in one clade of Firmicutes (Mollicutes) at the expense of Bacteroidetes, a collapse in overall diversity, and a metagenome enriched for the import and fermentation of simple sugars (7). When transplanted into lean germ-free recipients, this diet-induced community caused greater fat deposition, demonstrating that the altered microbiota is not a passive consequence of the diet but a functional trait that is transmissible (7). The same trend, a decreased diversity and a shift to pro-inflammatory, metabolic-risk-related taxa, can be seen in humans with obesity and metabolic syndrome (8). What we take forward is that the dysbiotic community built up by a Western diet is the community that, as section 4 demonstrates, bears a heavier burden of resistance genes.

## 3 Diet-driven cardiometabolic disease as a silent cause of AMR

This is the thinking part of this review. We hypothesized that obesity, T2D, hypertension, and CVD are not only associated with infection and antibiotic exposure but also increase AMR risk via convergent evidence-based pathways (Figure 1).

**Figure 1:**
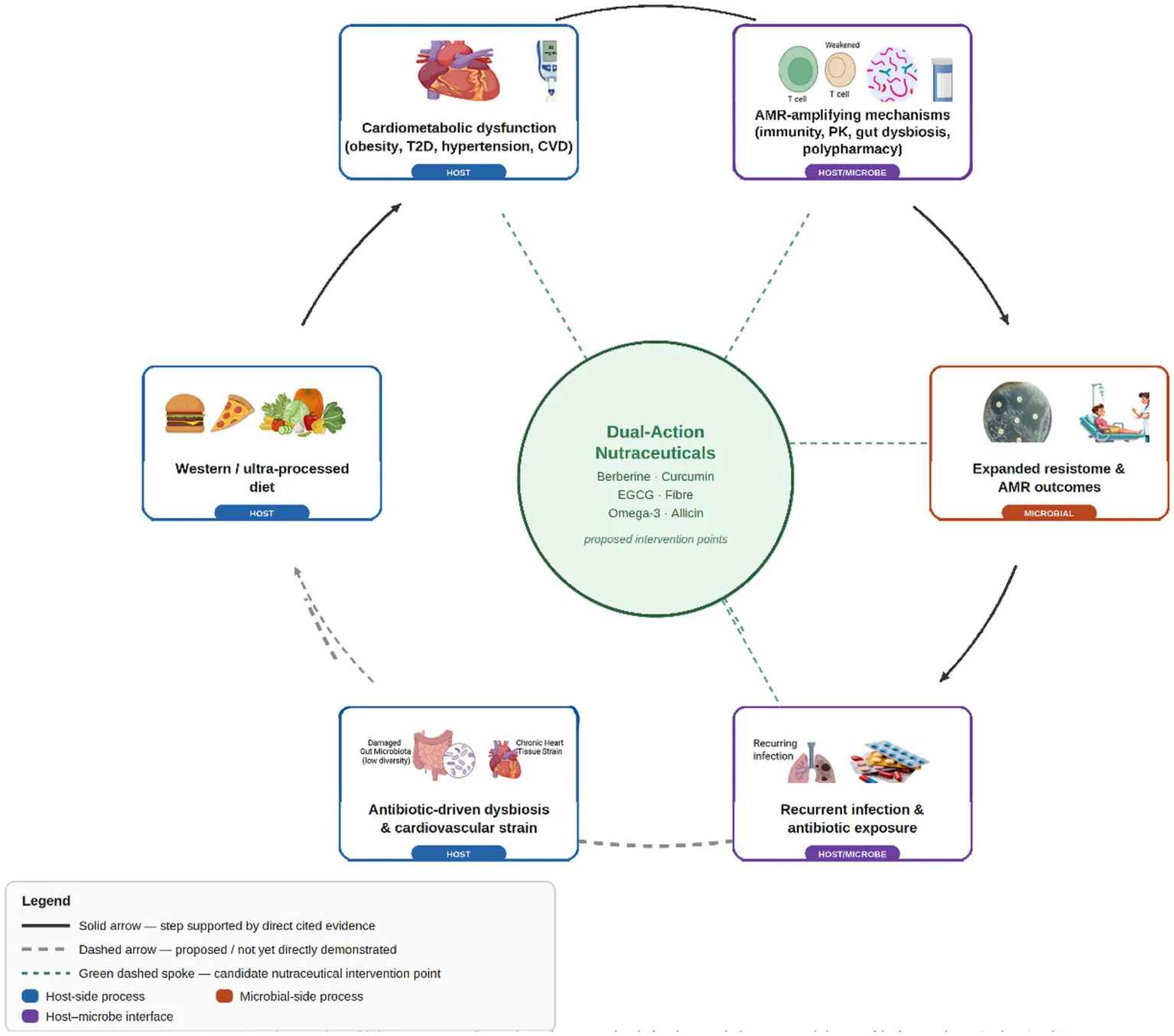
Metabolic–AMR augmentation loop. Cardiometabolic dysfunction is a consequence of the Western diet and can increase the risk of AMR via four pathways (immune impairment, pharmacokinetic underdosing, gut dysbiosis, and polypharmacy). This leads to resistant infections and the growth of a resistome, thereby increasing exposure to antibiotics, which in turn contributes to antibiotic-driven dysbiosis and higher cardiovascular risk, further compounding metabolic state. The central node (dual-action nutraceuticals) can be used to break the loop at the metabolic- and antimicrobial-effector level. Solid arrows indicate steps with explicit support from the cited studies, while dashed arrows indicate steps that are still proposed but not proven (the loop-closing steps from antibiotic exposure to worsening metabolic dysfunction are the most speculative). Colored tags are used to separate steps on the host side, the microbial side, and the interface.

### 3.1 Hyperglycemia and immune dysfunction connection

Neutrophils are damaged by poor glycemic control, as they are the first cells that a wound or bloodstream organism encounters. Hyperglycemia inhibits neutrophil chemotaxis, phagocytosis, and killing by oxidative burst, in part by activating protein kinase C, increasing the number of advanced glycation end-products, and non-enzymatically glycating cytoskeletal proteins involved in engulfment (13). Impairment is not a generalized suppression; rather, in the whole blood of individuals with type 2 diabetes, neutrophils are hyperactivated in the presence of hyperglycemia to produce reactive oxygen species (ROS) and neutrophil extracellular traps (NETs) through autophagy, and their phagocytosis and killing ability of *Streptococcus pneumoniae* is impaired when stimulated (13). Phagocytosis and the production of NETs share the same oxidative currency, indicating that diverting one resource does not necessarily result in the other. The pattern is glucose-dependent and reversible; high glucose levels alone are sufficient to trigger NETosis in cultured neutrophils and in patient plasma (14). Circulating NET markers are increased in poorly controlled type 2 diabetes and decrease upon achieving glycemic control (15). Diabetes predisposes neutrophils to excessive NET formation, which slows wound healing in mice and humans (16).

This cellular failure is exacerbated by the chronic inflammatory microenvironment of metabolic disease which dysregulates immune signaling more broadly (12). The everyday implication is that diabetics have a higher incidence of urinary tract, respiratory, skin and soft-tissue infections, but the crucial one for this argument is that it is resistant infections specifically that are more common in diabetics. People with type 2 diabetes were over two times as likely as those without to have antibiotic-resistant urinary infections (OR 2.42; 95% CI 1.83–3.20) and over two times as likely to have antibiotic-resistant respiratory infections (OR 2.35; 95% CI 1.49–3.69) as a meta-analysis of 61 studies and approximately 449,000 participants reported (17). Every infection event is another round of antibiotics; every round of antibiotics is another chance for resistance to be selected in the infected person and those they come into contact with.

### 3.2 Diabetic foot as a reservoir of multidrug-resistant organisms

Diabetic peripheral neuropathy and microangiopathy compromise the barrier function and deprive the wound bed of perfusion, leads to chronic diabetic foot ulcers (DFUs) that are colonized, resampled, and re-treated repeatedly. Today, these wounds serve as ecological reservoirs for multidrug-resistant (MDR) organisms and the scale is now well quantified. The prevalence of MDR organisms in DFUs was 50.86% (95% CI 41.92–59.78) with 1 infected ulcer in 2 patients, according to a meta-analysis of 21 studies and 4,885 patients, which included MDR organisms resistant to 3 or more antibiotic classes (18). The assumption that the wounds are dominated by staphylococci was overturned by the higher proportion of Gram-negative bacilli (32.84%) than the Gram-positive cocci (19.81%) that were resistant, with MDR *Escherichia coli* (6.93%) and MDR *Pseudomonas aeruginosa* (6.01%) being the leading resistant Gram-negative bacilli, and MDR *Staphylococcus aureus* (12.13%) the single most common resistant isolate overall (18, 19).

The same analysis associated MDR carriage with PV disease, peripheral neuropathy, nephropathy, osteomyelitis, a higher Wagner grade, previous hospitalization and previous antibiotic exposure, a pattern that is reminiscent of a history of long-standing, poorly controlled metabolic disease rather than a pattern of chance exposures (18). The burden is uneven: a Nigerian multi-center study (56) found the prevalence of MDR organisms across the etiology of DFUs was consistent with and in some locations higher than the global pooled estimate, highlighting the fact that the ulcers that are generating MDR are occurring in the lowest and middle income settings that have the least capacity to sustain the cost. But the resistance is not a laboratory curiosity, the MDR infection in these ulcers has been correlated with longer healing times, longer hospital stays and higher amputation rates (20). The diabetic foot is therefore not only a clinical burden, but the clearest worked example of the amplifier thesis, impaired host defense allows colonization, ischemia and neuropathy maintain the chronic wound, and the long, often empirical antibiotic courses that these wounds attract produce the very MDR flora that is recorded by surveillance.

### 3.3 Recurrent infection and cumulative antibiotic exposure

All of the mechanisms outlined above lead to a single quantitative variable that determines resistance, cumulative antibiotic exposure. Heavy burden of metabolic disease is associated with increased use of antibiotics because of increased infection rates, and because of the reduced clinical threshold for empirical antibiotic use due to the presence of comorbidity. The Danish national registries provided the numbers: community antibiotic prescriptions were 364 per 1,000 person-years in people with type 2 diabetes compared to 275 per 1,000 person-years in those without type 2 diabetes, with an adjusted rate ratio of 1.24 (95% CI 1.23–1.25) across urinary, skin and bloodstream infections (21). Resistance selection is related to the intensity and duration of exposure, so that a metabolically compromised population is, all else equal, a better engine of resistance than a metabolically healthy one (2).

#### 3.3.1 Pharmacokinetics of drugs in obese patients

At the same time, obesity affects the volume of distribution and clearance of numerous drugs: increased adipose mass increases the volume of hydrophilic drugs, increased renal plasma flow and glomerular filtration increase clearance, obesity-induced nephropathy or hepatic steatosis decreases clearance, and decreased perfusion of adipose tissue decreases drug availability at the infected site (22).

Body weight is not a reliable indicator of the direction of the net effect, which is why fixed standard dosing can be dangerous and why the detail is important for each class (23). Here the evidence is more selective than a blanket suggestion for higher doses. The consensus review of 128 pharmacokinetic studies found that the effect of obesity on β-lactams was modest and did not warrant routine dose adjustments, while for the aminoglycosides and glycopeptides the effect was significant enough to warrant weight-based dosing; for most other classes, the data were sparse and not reassuring, and therapeutic drug monitoring remained the safest approach (23). The current dosing recommendations take that into consideration and are represented in numbers: vancomycin is dosed based on total body weight, with the loading dose at the higher end of the recommended range; aminoglycosides are dosed based on ideal body weight, and only adjusted in cases where total body weight is greater than approximately 130% of the ideal body weight; and β-lactams are given at the upper end of the recommended doses, not increased uniformly (57). Individualization, not a fixed multiplier, is the appropriate answer, as in a population-pharmacokinetic model of obese adults (58), vancomycin clearance was related to lean body mass and renal function, and none of the available estimates of volume of distribution proved to be reliable. If the target exposure is reached, then the AUC/MIC ratio is approximately 400–600 based on covariate-guided dosing with monitoring. Be precise about what this evidence chain actually supports: the evidence for obesity-related pharmacokinetic changes is well established for vancomycin, aminoglycosides, and, to a lesser extent, β-lactams; direct clinical evidence of obesity-related pharmacokinetic changes contributing to the emergence of resistance, as opposed to underdosing and failure, is still limited to a few pharmacodynamic modeling and observational studies, and we will consider the two as related but separate claims throughout this review.

Under-exposure is the common failure mode of these agents and under-exposure is not benign, if the concentration of a drug is below its threshold for total suppression, but still within the range of the mutant selection window, then the resistant subpopulations are more likely to survive and expand, while the susceptible subpopulations are more likely to die out, and the patient with obesity dosed as if of average build is effectively conducting a small resistance-selection experiment at the site of infection. Obesity therefore represents a system and structure that predisposes to the very exposure profile that leads to resistance, regardless of whether there is a behavioral factor involved. The response to individual antibiotics is, in addition, further influenced by the interaction between diet, gut microbiota and host metabolism; diet and metabolic state therefore not only determine the presence of microorganisms, but also the range of concentrations that is encountered (24).

### 3.4 Hypertension, polypharmacy and the bidirectional antibiotic–cardiometabolic loop

There are two additional contributions: hypertension and established CVD. They increase the risk of exposure to infection and the cumulative use of antibiotics due to the increased risk of polypharmacy and frequent healthcare contact. Second, and more novel, the relationship seems to be bi-directional. A large-scale nationwide study of over 2 million adults showed that there was a small but dose-dependent link between cumulative antibiotic exposure and incident cardiovascular disease, potentially mediated by disruption of the gut microbiota by antibiotics (25). This is a closed loop, not a one-way street: cardiometabolic disease leads to antibiotic use and antibiotic use seems to lead to cardiometabolic disease (Figure 1). An upstream, metabolic intervention could have a leverage greater than what appears at first glance to be its distance from the microbiology, which is the whole point of the loop.

## 4 Gut resistome ecology

### 4.1 General dynamics of the gut resistome

The gut resistome is the sum of all the genes encoding antibiotics resistance (ARGs) found in the intestinal microbial ecosystem. These genes are densely retained in commensals and opportunistic pathogens of the gut and are continuously replenished from food, water and the environment and are enriched under antimicrobial selection (3). The ARGs are transmitted vertically, from parent to daughter cell, and horizontally from one bacterium to another by transformation, transduction, conjugation and membrane-vesicle fusion, allowing the spread of resistance to be much more rapid than by mutation alone (3). Diet comes to play as an ecological regulator: it creates the nutrient landscape and the community composition in which ARGs are acquired, lost, selected and exchanged.

Distinctively, the resistome is a genetic reservoir, the number and diversity of ARGs from the gut community. A larger or more diverse resistome is not necessarily clinically relevant antimicrobial resistance in an infecting organism; the former refers to genetic potential, the latter to a phenotype that actually affects treatment. The evidence that a change in resistome composition leads to more difficult-to-treat infections is less robust and more indirect, as the remainder of this section demonstrates. We indicate which of the two we are referring to at each step below.

### 4.2 Influence of diet on the gut resistome

There are several converging lines of evidence that diet affects the resistome, and the microbiota. Among healthy US adults, increased dietary fiber intake correlated with decreased ARG burden, while increased dietary protein intake, especially beef and pork, correlated with increased ARG burden and increased facultative anaerobes (Streptococcaceae and Enterobacteriaceae) (26). The resistome signature of long-term dietary patterns: comparisons of omnivores, pescatarians, vegetarians and vegans show differential abundances of specific ARGs, with some tetracycline-resistance genes being more abundant in omnivores (27, 28). The mechanism proposed is the ecological one of producing short-chain fatty acids from fiber by obligate anaerobes, which will reduce the luminal pH to favor the growth of anaerobes, as opposed to the facultative Enterobacteriaceae which are the primary carriers of transferable resistance (26). The resistome can thus be trimmed by modifying the community that hosts it. The relevance of the disease is highlighted by the fact that gut resistomes are expanded in disease states in general (29) and gut ARG profiles have been shown to have measurable global health risk (30).

### 4.3 The diabetes ARG score as a quantitative bridge

The strongest quantitative association between the resistome and metabolic disease is the one study that was well-powered and large. The composition of the gut antibiotic resistome was found to be significantly different among healthy, prediabetic and diabetic adults in a multi-omics cohort of 1,210 adults, with higher ARG diversity linked to increased risk of T2D, and a derived “diabetes ARG score” positively correlated with glycemic traits, and longitudinal validation of this score showed a correlation with changes in insulin resistance, which tracked the progression of T2D (9). This is a historic finding for the field, as it moves resistance genes not only alongside the cardiometabolic risk architecture, but inside of it: the resistome is not only a bystander in metabolic disease, but it is a measurable correlate of the progression of metabolic disease (9). Consistent observations are found in obesity, as obese people have higher ARG loads and a different Bacteroidetes/Proteobacteria ratio (31) and in various metabolic and inflammatory diseases in which particular ARGs are linked to disease-associated dysbiosis (29).

It is important to be honest about the extent of this evidence. The diabetes ARG score is a research score, not a validated clinical biomarker, and is not currently used in external clinical implementation; it has only been used in one cohort (the Guangzhou Nutrition and Health Study, a Chinese adult population) and has not yet been replicated in other cohorts or ethnicities. The associations are mostly correlational, but the one-sample Mendelian randomization analysis performed by the original study is a promising initial step to disentangling the two, and one in this field needs to be repeated with independent instruments and larger samples before the score can be considered more than a promising lead. An association between ARG diversity and glycemic traits is not necessarily an association between phenotypic drug resistance in individual patients and ARG diversity.

### 4.4 An ecological model of resistome evolution under dietary pressure

The results are consistent with an ecological model in which diet is a selective environment (Figure 2). A Western diet reduces the production of short chain fatty acids by fermentation of fiber, increases the pH of the lumen and increases the abundance of facultative anaerobes which are effective receivers and donors in horizontal gene transfer and are disproportionately associated with MDR gene clusters (3, 26). Transfer is further enhanced by non-antibiotic dietary and pharmaceutical stressors which increase horizontal transfer of ARGs by increasing the levels of reactive oxygen species, membrane permeability and DNA uptake (32). The resistome that develops is therefore a result of the diet-induced niche, not just antibiotic history, but a dietary intervention can shift the resistome (section 6).

**Figure 2:**
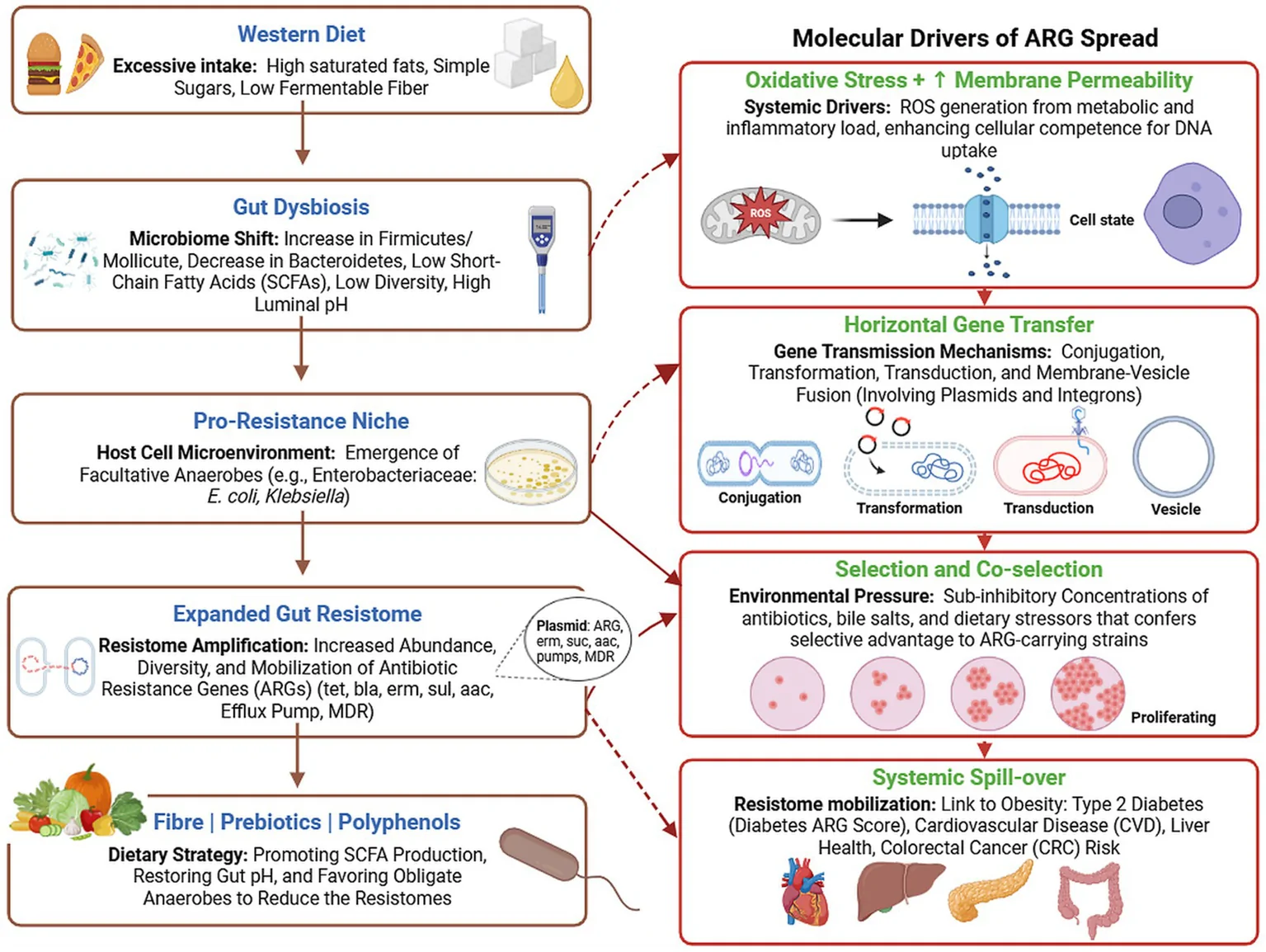
The gut resistome is influenced by diet. A Western diet leads to dysbiosis (decrease in the production of short-chain fatty acids, increased luminal pH, decreased diversity, increase in ARG richness and diversity) creating a niche for facultative anaerobes like Enterobacteriaceae. Molecular drivers (on the right) such as oxidative stress and membrane permeability, horizontal gene transfer, selection and co-selection, increase the rate of ARG spread, and the spread of the resistome reflects systemic metabolic risk. Fiber, prebiotics and polyphenols cut the chain by re-establishing the short-chain fatty acids and reducing the pH. Solid arrow indicates published studies directly show this step. Dashed arrow indicates a proposed step, which follows from the evidence but has not yet been shown directly in people. The dashed arrows mark two things. The first is the idea that the long-term oxidative stress seen in obesity and diabetes increases gene transfer inside the human gut, which so far has been shown only in laboratory systems. The second is the link from resistome changes to diabetes, heart disease and other conditions, which rests on one unreplicated cohort and is based on association, not proven cause. The gray dashed lines around the plasmid bubble show a close-up view. They are not a step in the process.

## 5 Molecular mechanisms of diet-AMR relationships

Underlying the ecology is a series of molecular events that drive diet-induced metabolic and inflammatory stress that leads to resistance. We highlight four that link the host state with bacterial fitness and gene flow.

### 5.1 Oxidative stress and horizontal gene transfer

The metabolic disease-associated chronic oxidative and inflammatory burden produces ROS that cause membrane damage, permeability, and stress response that promote foreign DNA uptake and transfer. Non-antibiotic stressors have been demonstrated to promote ARG transfer via this mechanism: ROS overproduction, increased membrane permeability and increased competence, which promotes conjugation, transformation and transduction in the gut (32). That demonstration comes from defined experimental systems, however, and whether the lower-grade, sustained oxidative stress of obesity or T2D nudges the same machinery *in vivo* is at this point a plausible extrapolation from that experimental biology, rather than a directly observed pathway, and we present it as such.

Diet also interrupts and influences microbial structure via nutrient accessibility. Regulation of metabolic state and stress responses, shifting antibiotic uptake and tolerance (33). These diet-driven shifts in metabolism and environment in turn modify horizontal gene transfer (HGT). Fermentable fiber-derived short-chain fatty acids (SCFAs) acidify the gut and ease plasmid conjugation, while diet-induced inflammation (oxygenation) endorses plasmid and phage-mediated ARG propagation (33).

### 5.2 Efflux pumps

Active efflux is a major, but frequently overlooked resistance mechanism, and an efflux of structurally diverse drugs reduces intracellular levels. Efflux systems are also weak spots: several food bioactives are able to inhibit them, and to re-sensitize MDR organisms, which is the basis of the adjuvant strategies discussed in sections 6 and 8 (34–36).

### 5.3 Biofilms and quorum sensing

Bacteria use quorum sensing to control virulence and biofilm programs, and biofilm formation contributes to protection against antibiotics and immune clearance, and to a physically dense environment for gene exchange. Compounds that inhibit biofilm and quorum sensing would thus lower virulence and the local rate of resistance transfer (37). Linking this mechanism to chronic wounds and mucosal surfaces of metabolic disease, these are favorable biofilm habitats.

### 5.4 Subtherapeutic coverage as a selective sieve

Lastly, the obesity pharmacokinetics of section 3.3.1 act as a molecular selection sieve: drug concentrations within the susceptible and resistant inhibitory thresholds preferentially favor resistant subpopulations. Diet and metabolic state therefore influence both the presence of organisms and the concentration window to which they are exposed (23, 24).

## 6 Nutraceuticals as dual-action AMR interventions

If diet-induced metabolic dysfunction exacerbates AMR, then interventions that restore metabolic function should remove resistance pressure at its source. The most interesting compounds are those that target both arms of the nexus simultaneously: they have a beneficial effect on metabolic health and also have a direct antimicrobial or anti-virulence/resistome modulatory activity. First we introduce the best example and then we look at the rest of the class (Table 3).

**Table 3:** The summary of the evidence for the dual-action nutraceuticals mentioned in section 6.

Compound	Evidence level	Target organism(s)	Proposed mechanism	Observed AMR-relevant effect
Berberine	*In vitro* + rodent; no human AMR trials	*Escherichia coli* (FtsZ); broad MDR Gram-positive/negative via efflux modulation	AMPK activation (metabolic side); FtsZ/Z-ring inhibition and efflux-pump blockade (antibacterial side)	Restores antibiotic susceptibility in MDR isolates *in vitro*; glycemic benefit shown in humans, antimicrobial benefit not yet tested in humans
Curcumin	*In vitro* + rodent	*Acinetobacter baumannii*; methicillin-resistant *Staphylococcus aureus*	Anti-biofilm activity; lowers MIC of partner antibiotics	Biofilm inhibition and MRSA re-sensitization *in vitro*; poor oral bioavailability limits translation
EGCG (green tea catechin)	*In vitro*	Broad Gram-positive/negative	Membrane disruption; interferes with DNA supercoiling	Potentiates gentamicin *in vitro*
Cranberry proanthocyanidins	*In vitro*	Staphylococci	Beta-lactamase inhibition	Re-sensitizes MRSA to beta-lactams *in vitro*
Rosemary terpenes	*In vitro*	Efflux-expressing strains	*NorA* efflux-pump inhibition	Potentiates erythromycin *in vitro*
Matrine	*In vitro*	*Pseudomonas aeruginosa*	Efflux and biofilm inhibition	Re-sensitizes MDR *P. aeruginosa* to fluoroquinolones *in vitro*
Dietary fiber/prebiotics	Human intervention (pediatric cohort)	Gut Enterobacteriaceae and other ARG-carrying taxa	SCFA-mediated luminal acidification; favors obligate anaerobes over facultative anaerobes	Reduced gut ARG richness/diversity together with improved metabolic syndrome in the same children; the strongest dual-outcome human evidence in this review
Probiotics	Human; strain- and person-specific	Gut MDR pathogens	Competitive exclusion; antimicrobial-peptide production	Inconsistent: can lower or raise ARG abundance depending on strain and individual
Omega-3 fatty acids	Human RCTs (cardiovascular endpoints); resistome/AMR evidence indirect	Not organism-specific	Reduces systemic inflammation (host side); may upregulate bacterial efflux in some settings	Metabolic/anti-inflammatory benefit well evidenced in humans; direct antimicrobial or resistome effect not established

### 6.1 Dual action berberine as the reference molecule

The single molecule that is most clearly a manifestation of the dual-action concept is berberine, an isoquinoline alkaloid found in goldenseal, *Coptis* species and *Berberis* species (Figure 3). On the metabolic arm, berberine activates AMPK, the central metabolic sensor of the cell, which in turn enhances glucose handling and insulin sensitivity, and inhibits the production of the inflammatory molecules TNF-α, IL-1β, IL-6, MCP-1, iNOS and COX-2 by adipose-tissue and macrophages, thus reducing the metaflammation involved in metabolic disease (38). Berberine binds to the bacterial cell-division protein FtsZ with high affinity, prevents assembly of the Z-ring, and inhibits GTPase activity of FtsZ, preventing cytokinesis, a mechanism that is not known to result in cross-resistance with any class of front-line antibiotics (39). Berberine and related protoberberine alkaloids also block bacterial efflux pumps and enhance the activity of conventional antibiotics against MDR bacteria, and they alter the gut microbiota, which could plausibly affect the resistome (34). Thus, a single molecule reduces the metabolic incentive to use antibiotics and directly impacts bacterial fitness and virulence, the synthesis that gave birth to this review.

**Figure 3:**
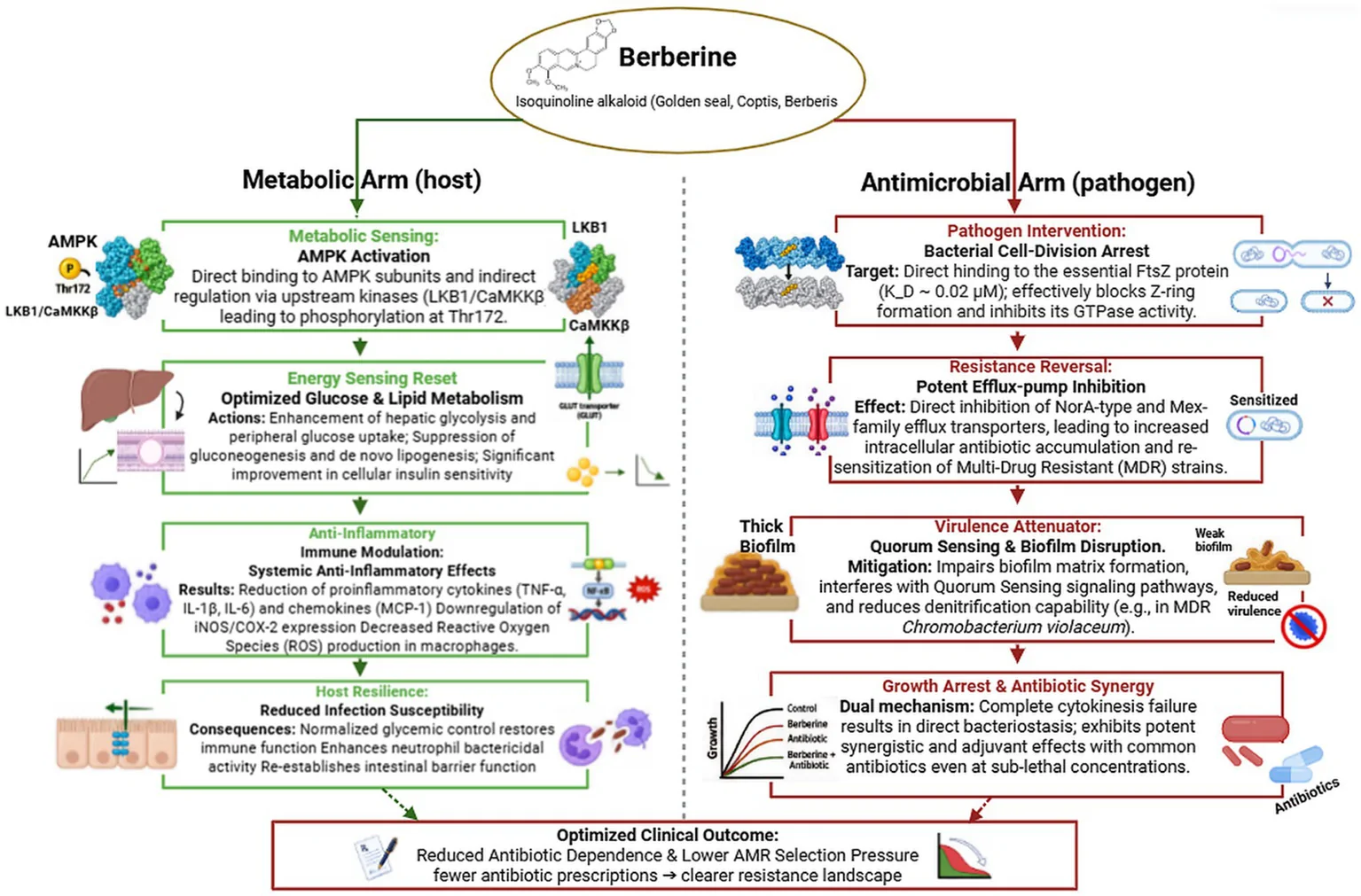
Berberine has an effect on both sides of the diet—AMR connection. Left (host/metabolic arm): berberine is a stimulator of AMPK, and resets energy metabolism, increases insulin sensitivity, and decreases inflammatory cytokine production, thus decreasing the burden of infection. Right (pathogen/antimicrobial arm): berberine inhibits FtsZ dependent cell division, efflux pumps, and biofilm, quorum sensing and virulence. Both arms result in reduced antibiotic dependence and less AMR selection pressure. Solid arrow indicates that published studies directly show this step. The dashed arrow is a proposed step. It follows from the evidence but has not yet been shown directly in people. Every effect of berberine on the host and on bacteria is supported by direct studies, most of them in cells or animals. The final step, which these effects lead to less antibiotic use and lower resistance pressure in patients, is dashed for two reasons. No human trial has yet measured this outcome. Also, berberine is poorly absorbed when taken by mouth, so the levels reached in the body may be lower than those used in the laboratory tests.

The majority of the mechanistic and antibacterial results presented above are derived from *in vitro*, cell-culture, and rodent-based studies and have not been conducted in a randomized human trial setting nor tested against an AMR or resistome endpoint. This is a real challenge: oral bioavailability of berberine is low and variable and typically reported to be less than 5%; thus, levels in plasma and tissues after a typical metabolic dose would not be expected to reach the levels used in the previously mentioned antibacterial assays. It is generally well tolerated at doses used for glycemic control, although gastrointestinal side effects are common, and interacts with CYP3A4- and CYP2D6-metabolized drugs; its safety at higher or more sustained exposures as might be required for an antimicrobial application is simply not known. We are introducing berberine as the best example of a dual-action therapy not one that is ready to use for that purpose.

### 6.2 Curcumin

The main polyphenol of turmeric is curcumin which has anti-inflammatory and metabolic properties, and also has direct antimicrobial activity, although it varies between strains and in general is more effective against Gram-positive than Gram-negative microorganisms (40). It is significant for resistance because of two properties, which are not just growth inhibition properties. First, it is an anti-biofilm agent: Of the 12 flavonoids tested against *Acinetobacter baumannii*, curcumin was the most active, inhibiting biofilm formation by reference and multidrug-resistant clinical isolates better than the reference inhibitor gallium nitrate, and inhibiting pellicle formation and surface motility as well (41). Second, it restores the activity of failing antibiotics: when combined with ampicillin, oxacillin or norfloxacin, curcumin caused synergistic decreases in the minimum inhibitory concentration against methicillin-resistant *Staphylococcus aureus* (MRSA), making it sensitive to antibiotics it was resistant to (42). Similarly, like berberine, the major hurdles in translation are its poor aqueous solubility and bioavailability, and hence, nanoformulation and co-formulation with berberine is actively being pursued; the alkaloid-polyphenol pair (berberine and curcumin) has been co-encapsulated in targeted liposomes with high encapsulation efficiency (43) (section 8). However, despite its long history as a safe dietary supplement, this evidence base is predominantly preclinical, with the antimicrobial potential of curcumin being a laboratory rather than a clinical signal, given its own suboptimal oral bioavailability and rapid first-pass metabolism, as well as the lack of any trial powered for an AMR or resistome outcome. The dual-action property, metabolic and antimicrobial activity, is not unique to berberine but is a broader class of compounds for which translation, rather than discovery, is the limiting step.

### 6.3 Catechins (EGCG)

The dominant green-tea catechin, epigallocatechin gallate (EGCG), disrupts bacterial membranes, interferes with DNA supercoiling and has been shown to have significant synergy with aminoglycosides, with EGCG plus gentamicin being effective in killing MDR *Staphylococcus aureus* and *Escherichia coli* and preventing resistance development, in addition to its established metabolic and antioxidant properties (36).

### 6.4 Other polyphenols and phytochemicals

The broader phytochemical literature confirms the pattern. Persimmon tannin is responsible for the suppression of MRSA by disrupting membrane potential and protein integrity (44); cranberry proanthocyanidins inhibit beta-lactamases and re-sensitize MRSA to oxacillin and carbenicillin (45); terpenes from rosemary inhibit the efflux pump NorA and potentiate erythromycin (3) and alkaloids like matrine inhibit efflux and biofilm formation and re-sensitize MDR *Pseudomonas aeruginosa* to fluoroquinolones (35). Whereas the metabolic evidence is not as robust as for berberine, many of these compounds also possess an anti-inflammatory activity, making them potential dual-action compounds.

### 6.5 Dietary fiber, prebiotics and the resistome

The ecological pathway by which fiber and prebiotics impact the resistome is the one described in section 4. Fiber is fermented in the gut to produce short-chain fatty acids, which lower the pH of the gut lumen and increase the abundance of obligate anaerobes, while reducing the abundance of facultative anaerobes, Enterobacteriaceae, which carry most of the transferable resistome. The compelling evidence is the diet-based gut microbiota intervention in obese children using a whole-grain-based diet with prebiotics that resulted in a significant decrease in the richness and diversity of the gut resistome, including the elimination of dozens of ARGs and the reduction of ARGs-encoding Klebsiella, Enterobacter, and Escherichia species, and a simultaneous improvement in metabolic syndrome in the same children (46, 47). This is the single, dual outcome piece of evidence in the entire review: one dietary intervention, two simultaneous benefits, and contraction of the resistome and metabolic improvement in the same cohort.

### 6.6 Omega-3 fatty acids and probiotics

The metabolic/anti-inflammatory arm of omega-3 fatty acids is the primary mechanism, as it decreases systemic inflammation, which compromises host defense, and their direct effect on bacterial resistance is more complicated and, in some settings, may promote the upregulation of efflux pumps; hence, they are best considered as upstream regulators rather than direct antimicrobials (3). Probiotics can inhibit the growth of MDR pathogens by competitive exclusion and production of antimicrobial compounds, and reduce ARG abundance; however, this is also a double edged sword as probiotic strains can contain ARGs that can be transferred to other bacteria and probiotic intake can increase ARG abundance in some individuals due to horizontal transfer (48). The conservative interpretation is that probiotics and omega-3s should be used in combination and not as standalone treatments, that the effects on the resistome are not consistent across all probiotic strains, and that the effects are not consistent across individuals (the same probiotic strain can reduce ARG abundance in one person and have no effect in another and can even increase abundance in another). The evidence to date does not support blanket recommendations for AMR mitigation.

## 7 Omega-3 and omega-6 fatty acids dietary balance, inflammation, and AMR relevance

Omega-6 and omega-3 polyunsaturated fatty acids are dietary fats that influence cell membranes, lipid metabolism, immune function, and cell signaling (49). The major dietary source of omega-6 is linoleic acid (LA), while alpha-linolenic acid (ALA), EPA, and DHA are the most clinically relevant omega-3s (49). In the case of canned sardines, for example (59), the two families of fatty acids sit side by side in the same tin, as this is as good a reason as any to ask what their balance does to health, instead of assuming one is “good” and the other “bad.”

The widely held notion that dietary omega-6 is pro-inflammatory is not supported by the evidence from trials. Johnson and Fritsche conducted a systematic review of RCTs that showed no consistent effect on CRP, fibrinogen, cytokines, or TNF-α (50), while Wang et al. (51) review of 40 RCTs (1,377 participants) also concluded that there was no consistent effect on CRP and IL-6. Despite this, Li et al.’s meta-analysis of 31 RCTs did show that reducing the omega-6 to omega-3 ratio resulted in a decrease in levels of TNF-α and IL-6 but not CRP (52), which suggests that the ratio has some biological signal but is not a clean clinical marker. For cardiovascular effects, Farvid et al. (61) did a meta-analysis of 13 cohorts (310,602 participants) and determined that the highest vs. lowest LA intake was associated with a 15% decrease in the incidence of CHD and a 21% decrease in coronary mortality; a Cochrane review of long-chain omega-3 intake by Abdelhamid et al. (53) and a meta-analysis of 38 RCTs (149,051 participants) by Khan et al. (54) found a reduction in coronary events and mortality at the expense of increased risk of atrial fibrillation at higher doses. None of this is to demonize omega-6 or to suggest that omega-3 supplements are a sure bet, but both results are dose- and context-dependent, and the sardines-in-sunflower-oil tin, as it turns out, is still a good source of long-chain omega-3s, whatever oil it sits in (59, 60).

The point of including this in an AMR review, and not a nutrition digression, is the same as the one outlined in section 6.6. The omega-3/omega-6 balance has a primary effect on the host side of the loop. Lower-inflammation dietary patterns are plausibly antimicrobial immune competent and infection-resolving; neither fatty-acid family has good evidence of direct antimicrobial activity, and in some settings omega-3s have even been associated with increased bacterial efflux activity (section 6.6). Omega-3/omega-6 balance is included as a potential upstream mechanism to enhance host defense and reduce the need for antibiotics, but not as an anti-resistance nutraceutical.

## 8 The application of more than one drug and delivery system

The compounds listed above are most useful not as single drugs but as adjuvants to antibiotics. They inhibit efflux, disrupt biofilm or damage the membrane, reducing the effective concentration of a partner antibiotic and can restore activity against otherwise resistant strains (34–36, 45). Examples include cranberry proanthocyanidins’ ability to re-sensitize staphylococci to beta-lactams (45), and EGCG’s ability to potentiate gentamicin (36) as well as protoberberine alkaloids’ ability to restore efflux-compromised drugs (34). There are two themes of translation. First, these adjuvant effects are frequently seen at sub-lethal concentrations and through anti-virulence rather than bactericidal mechanisms; this hypothesis, which is yet to be clarified, should be explicitly tested as it may create less resistance selective pressure than conventional bactericidal monotherapy. Second, the problem is delivery—both berberine and curcumin have poor oral bioavailability, and a variety of nano- and liposomal formulations are being developed to address these bioavailability limitations, such as curcumin–berberine co-formulations. However, delivery science is not an add-on or a side-note to the realization of the dual-action strategy; which is the main theme.

## 9 Precision nutrition against AMR

The variability of microbiota composition, resistome content, drug pharmacokinetics and dietary response of the individual throughout this review calls for precision, not a one-size-fits-all approach. Each person’s gut microbial community is unique, and antibiotic and dietary interventions have very different effects, as one person’s resistome is impacted by the same intervention and another’s is hardly affected (24). A precision nutrition program against AMR would involve metagenomic resistome profiling, host metabolic phenotyping and, in the future, machine learning models that could predict the dietary or nutraceutical intervention that will best lower the ARG load and metabolic risk of a specific person. The diabetes ARG score is a proof of concept that resistome features can be quantified and associated with metabolic trajectory (9) and the next step is to extend the score to include measures of intervention-outcomes.

There is no standardized approach to resistome profiling by shotgun metagenomics, and the same sample can contain different numbers of ARGs depending on the pipeline that is used; the composition of the gut microbiome is highly variable between people and even within people over time, so a single snapshot measurement of the microbiome is not a reliable basis for a personal recommendation; there are, as yet, no validated external datasets to test a precision-nutrition-for-AMR model; and, most fundamentally, there is no agreement in the field about what a clinically meaningful outcome looks like here, whether it is a reduction in ARG load, a reduction in infection frequency, or a reduction in treatment failure due to resistant infections. Machine learning models are only as accurate as the harmonized and validated data that are put into them, and this data is not yet available on the scale that this vision would require.

## 10 Public health translation dynamics

It is fair to suggest that patients with diabetes, obesity, hypertension, and CVD should be targeted for AMR priority in stewardship programs and that measures to prevent infection, optimize doses, and correct metabolic status should be considered as possible interventions, although we are not suggesting these interventions should be mandated by the current evidence base.

This is in line with the One Health approach to AMR and the sustainable-development agenda of healthy lives (1, 55). The following are a few translation points. Metabolic risk could be integrated into the logic of antimicrobial stewardship programs, combining dose optimization advice for obese patients with nutraceutical-adjunct and dietary-correction regimes. Bioactives from agro-industrial by-products provide a circular-bioeconomy solution that is appealing when costs are the key consideration, but will need to overcome the challenges of bioavailability, formulation, standardization and regulatory classification. The equity dimension is highlighted: metabolic disease and AMR disproportionately affects low and middle-income countries. While Monteiro et al. reported that the trend of sales expansion of ultra-processed products has clearly moved toward middle-income settings (11), Mozaffarian pointed out that reversing this trend is a food-environment and policy challenge rather than a clinical one (10). Lower cost food-derived interventions might thus be of special interest in areas with less advanced stewardship infrastructure. This is indicated as a hypothesis that should be formally health economically evaluated rather than a conclusion.

Before this reaches policy, one more difference should be made clear: infection burden reduction and AMR reduction are not the same. Reducing the opportunities for exposure will almost inevitably reduce the number of antibiotic courses that people receive, as will metabolic correction of reducing the rate of infection. Whether it also decreases the prevalence of resistant organisms in a population is a more difficult question, which relies on the dynamics of transmission, on stewardship outside the system, and on the difference between resistome and AMR, as explored in section 4. This review would make us recommend to those planning a study or policy to be explicit from the beginning which of the two outcomes they are seeking to influence.

## 11 Information gaps and prospective directions

We propose a framework as a hypothesis, and the most obvious research priorities are the weakest links. There are 5 gaps that stand out. First, there really are no longitudinal cohorts that track diet, metabolic state, and the composition of the resistome over time. Second, and most importantly, there are no randomized trials with a dual primary endpoint: one metabolic outcome (HbA1c or HOMA-IR) and another infection frequency or the composition of the resistome. The intervention of whole-grain and prebiotic in obese children is an example of a feasible design along these lines and should be the template. Third, a lack of standardized dosing and formulation information for these dual-action compounds in metabolic populations means that at this time, no clinical recommendation can be made. Fourth, causality is the main problem in the field: nearly all the results in this review are observational, antibiotic use is a strong potential confounder that is often not fully addressed, and host genetics is scarcely investigated as an alternative explanation to diet. The use of Mendelian randomization, including the one-sample analysis already performed with the diabetes ARG score (9), is a promising approach to begin disentangling cause from correlation and should be widely applied throughout this literature, and geographic variation in resistome ecology is under-explored and relevant directly to the translation of these ideas into antimicrobial stewardship in low- and middle-income countries where both the metabolic-disease burden and the AMR burden are growing fastest. Fifth, the notion that anti-virulence and microbiome-modulating mechanisms are less selective for resistance than bactericidal mechanisms is attractive but must be tested as opposed to assumed.

## 12 Conclusive and testable hypothetical context

We have proposed that diet-induced cardiometabolic dysfunction may be a hidden contributor to antimicrobial resistance and that this may occur in an innocuous circle. Western diet, metabolic dysfunction and immune dysregulation, gut dysbiosis, increased infection burden, increased antibiotic exposure, selection of resistance, and further microbiome disruption, which leads to progressive cardiometabolic disease (Figure 1). The intervention node is located within the loop, not outside it. Dual-action nutraceuticals, such as berberine, can target the loop twice: on the driver side, they restore metabolic homeostasis, and on the effector side, they have anti-virulence and direct antimicrobial activity, both of which reduce antibiotic dependence and antibiotic resistance selective pressure (Figure 3). The synthesis redefines a chronic-disease problem as a communicable-resistance problem and, in the process, proposes metabolic correction as a public health strategy not yet taken up as an AMR-mitigation strategy. The evidence presented here is adequate to support the hypothesis and to define the experiments that should be performed to test it but not yet adequate to rule it out. It is a good place to stop a hypothesis and theory discussion and start the studies that should follow.

Current evidence favors a food-based and dietary-pattern approach rather than an omega-6-versus-omega-3 approach. The practical implication is not that omega-6-rich foods should be avoided, but that diets should contain adequate sources of EPA and DHA, particularly fatty fish (60), while maintaining an overall pattern rich in nutrient-dense foods. For sardines packed in sunflower oil, the scientific evidence therefore supports viewing the product as a valuable source of omega-3 fatty acids (59) while considering the added oil within the context of the person’s total dietary intake.

## Glossary

AHA: American Heart Association
ALA: α-linolenic acid
AMPK: AMP-activated protein kinase
AMR: antimicrobial resistance
ARG (ARGs): antibiotic resistance gene(s)
AUC: area under the (concentration–time) curve
CI: confidence interval
COX-2: cyclooxygenase-2
CRP: C-reactive protein
CVD: cardiovascular disease
DASH: Dietary Approaches to Stop Hypertension
DFUs: diabetic foot ulcers
DHA: docosahexaenoic acid
DNA: deoxyribonucleic acid
EGCG: epigallocatechin gallate
EPA: eicosapentaenoic acid
FtsZ: filamenting temperature-sensitive protein Z (bacterial cell-division protein)
GTPase: guanosine triphosphatase
HbA1c: glycated hemoglobin (hemoglobin A1c)
HDL: high-density lipoprotein (cholesterol)
HGT: horizontal gene transfer
HOMA-IR: Homeostatic Model Assessment of Insulin Resistance
IKKβ: inhibitor of nuclear factor-κB kinase subunit β
IL-1β: interleukin-1 beta
IL-6: interleukin-6
iNOS: inducible nitric oxide synthase
JNK: c-Jun N-terminal kinase
LA: linoleic acid
LDL: low-density lipoprotein (cholesterol)
M1: M1 (classically activated) macrophage phenotype
MCP-1: monocyte chemoattractant protein-1
MDR: multidrug-resistant (multidrug resistance)
MIC: minimum inhibitory concentration
MRSA: methicillin-resistant *Staphylococcus aureus*
NCD: non-communicable disease
NET/NETs/NETosis: neutrophil extracellular trap(s)/the process of NET formation
NF-κB: nuclear factor-κB
NIH: National Institutes of Health
NLRP3: NOD, LRR- and pyrin domain-containing protein 3 (inflammasome)
ODS: Office of Dietary Supplements
OR: odds ratio
PUFAs: polyunsaturated fatty acids
PV: peripheral vascular (disease)
RCTs: randomized controlled trials
ROS: reactive oxygen species
SCFAs: short-chain fatty acids
T2D: type 2 diabetes
TLR4: Toll-like receptor 4
TNF-α: tumor necrosis factor-alpha
